# Cutting for Stone: A Case Report of Isolated Pleural Silicosis in an Engineered Stone Fabrication Worker

**DOI:** 10.1016/j.chpulm.2025.100188

**Published:** 2025-06-20

**Authors:** Matthew Federbush, Alain Borczuk, Arunabh Talwar, Julissa Jurado, Abhinav Agrawal

**Affiliations:** a Division of Pulmonary, Critical Care, and Sleep Medicine, Donald and Barbara Zucker School of Medicine at Hofstra/Northwell, Hempstead, NY; b Department of Pathology and Laboratory Medicine, Long Island Jewish Medical Center, Northshore University Hospital, Hempstead, NY; c Pathology and Laboratory Medicine, Donald and Barbara Zucker School of Medicine at Hofstra/Northwell, Hempstead, NY; d Advanced Lung Disease Program, Pulmonary Function Laboratory and Rehabilitation, Division of Pulmonary, Critical Care, and Sleep Medicine, Manhasset, NY; e Institute of Health System Science, Feinstein Institutes for Medical Research, Hempstead, NY; f Donald and Barbara Zucker School of Medicine at Hofstra/Northwell, Hempstead, NY; g Department of Thoracic Surgery, Donald and Barbara Zucker School of Medicine at Hofstra/Northwell, Hempstead, NY; h Interventional Pulmonology, Northwell Health Lung Institute, Hempstead, NY; i Cardiovascular and Thoracic Surgery, Donald and Barbara Zucker School of Medicine at Hofstra/Northwell, Hempstead, NY

**Keywords:** engineered stone, lymphadenopathy, occupational lung disease, pleural disease, silicosis

## Abstract

Silicosis typically presents with parenchymal lung disease in workers exposed to silica. We present a rare case of isolated pleural and lymph node silicosis without parenchymal involvement in a stone fabrication worker. A 49-year-old man with extensive occupational exposure to stone materials, including engineered stone without consistent use of personal protective equipment, was found to have mediastinal and hilar lymphadenopathy. Initial bronchoscopic and radiology evaluations were nondiagnostic. Video-assisted thoracoscopic surgery revealed pleural nodules, and pathologic examination demonstrated silicotic changes in both pleura and lymph nodes but without parenchymal involvement. This case demonstrates an unusual presentation of silicosis confined to the pleura and lymph nodes, highlighting the importance of thorough evaluation of thoracic pathology in workers with silica exposure. It adds to growing evidence regarding health risks in the engineered stone fabrication industry and emphasizes the need for improved occupational safety measures and medical surveillance.

Silicosis is an occupational lung disease caused by silica crystal inhalation. Acute silicosis develops after large exposure over the span of weeks or years with dyspnea, cough, and weight loss and diffuse ground glass opacities on imaging. Chronic silicosis may present with cough and dyspnea, manifesting as either nodular disease or progressive massive fibrosis.^1^ Pleural involvement is common but is extremely rare without parenchymal involvement.^2^ We present an unusual case of silicosis isolated to the pleura and lymph nodes (LNs).

## Case Report

**Figure 1:**
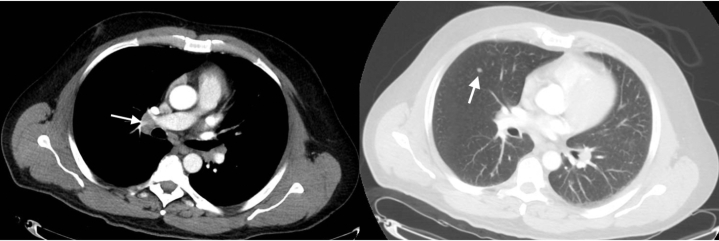
A, B, Trauma protocol CT angiography of chest. A, Mediastinal window with mild mediastinal and hilar lymphadenopathy (arrow – right level 10 lymph node). B, Lung window with 5-mm right major fissure nodule (arrow).

A 49-year-old man with a prior smoking history (4 pack-years) and extensive stone fabrication experience presented to the hospital after a fall. Incidental CT findings revealed mediastinal, hilar, and celiac plexus lymphadenopathy with stable pulmonary nodules but no interstitial, airway, or pleural disease (Fig 1). Detailed occupational history included working with natural (eg, marble, granite) and engineered stone materials, with rare access to reliable personal protective equipment or environmental controls. Review of systems included occasional hot flashes, night sweats, and hand discomfort.

Initial bronchoscopy with endobronchial ultrasound and transbronchial needle aspiration of subcarinal LNs showed only reactive LNs, with bronchoalveolar lavage (growing *Mycobacterium avium* complex [MAC]). PET scan revealed fluorodeoxyglucose-avid bilateral mediastinal, hilar, and celiac lymphadenopathy. Laboratory evaluation revealed mildly elevated nonspecific inflammatory markers with negative rheumatologic workup. After repeat PET scan showed increased avidity in the celiac LN, a CT-guided needle aspiration was performed but proved nondiagnostic. Because of persistent diagnostic uncertainty, a video-assisted thoracoscopic surgery with mediastinal LN dissection was performed. Intraoperative examination revealed innumerable tiny visceral and parietal pleural nodules, prompting pleural biopsy and left lower lobe wedge resection (did not include known lung nodules).

**Figure 2:**
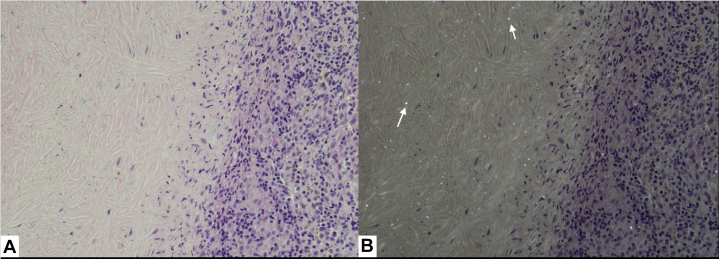
A, B, Example of lymph node histopathology. A, Hematoxylin and eosin stain (×200 magnification). B, Same sample under polarized light demonstrating silica crystals (arrows).

**Figure 3:**
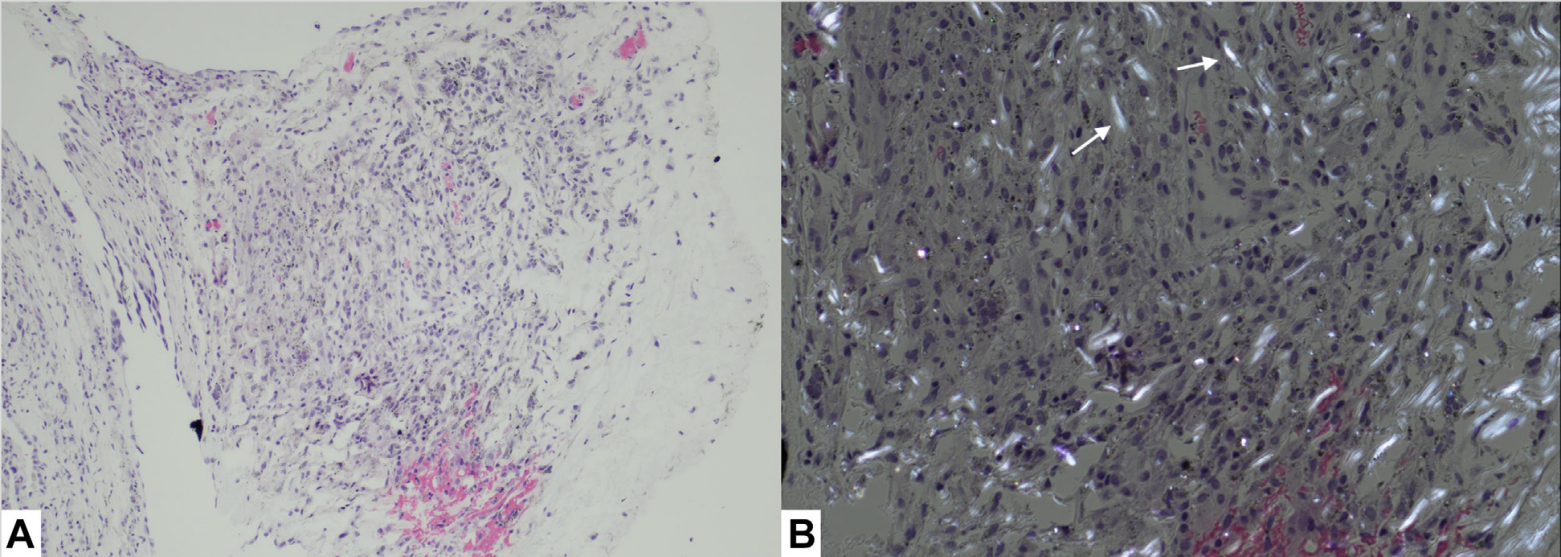
A, B, Example of visceral pleura nodule histopathology. A, Hematoxylin and eosin stain (×200 magnification). B, Same sample under polarized light demonstrating silica crystals (arrows).

**Figure 4:**
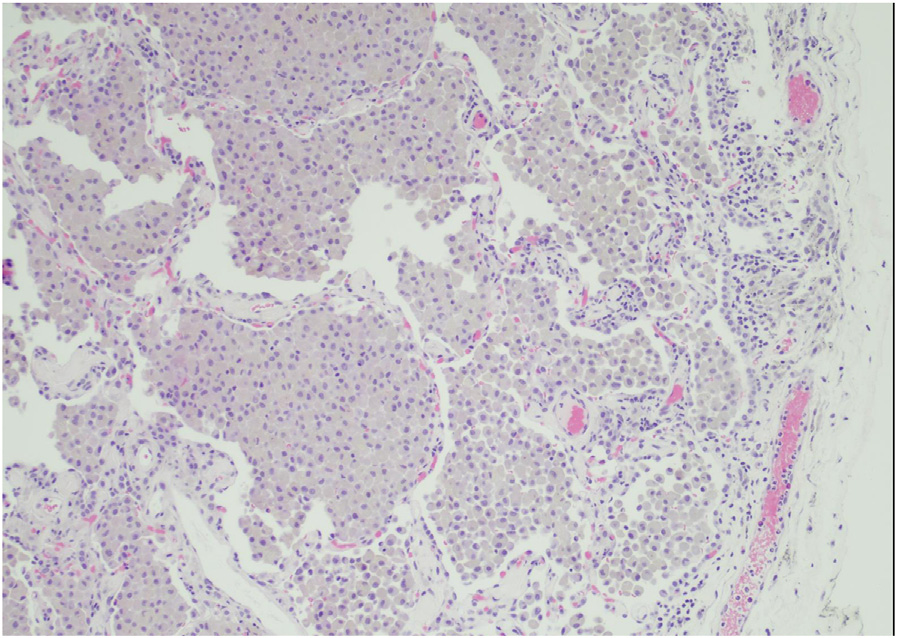
Example of Hematoxylin and eosin staining (×200 magnification) of lung parenchyma demonstrating respiratory bronchiolitis.

Pathologic examination of the LNs demonstrated hyalinized areas consistent with mixed dust exposure, including silica (Fig 2). The pleural biopsy had small histiocytic aggregates with a mild increase in polarizable material, consistent with silicotic nodules (Fig 3). Wedge biopsy revealed intraalveolar accumulation of pigmented macrophages, mild interstitial thickening, and airways remodeling, consistent with respiratory bronchiolitis (Fig 4).

## Discussion

Pleural involvement is common in silicosis,^2^ with CT scans frequently revealing pleural thickening, and occasionally pleural pearls,^3^ but rarely in absence of parenchymal disease.

Xeren et al^4^ described a 57-year-old plumber with silica exposure who presented with pleural thickening and small effusion with biopsies showing spindle-shaped macrophages containing birefringent silica and rare silicotic nodules. No other cases of isolated pleural silicosis were found in a literature review.

In this case, MAC infection and smoking history were initially considered, but the patient's imaging findings, symptoms, and significant silica exposure pointed away from these diagnoses and toward occupational lung disease.

The patient had 2 stable pulmonary nodules (5 and 6 mm), likely fissural LNs. Although pleural and LN specimens showed silicosis, lung tissue revealed only respiratory bronchiolitis. This absence of parenchymal silicosis represents an exceptionally rare presentation. Notably, pleural involvement was undetectable on CT scan and only discovered during video-assisted thoracoscopic surgery. Retrospective examination of celiac LN specimens showed rare polarizable material, whereas endobronchial ultrasound and transbronchial needle aspiration samples were acellular, potentially because of silica-related sclerosis.

This patient’s MAC positivity also deserves attention because he lacked typical risk factors and evidence of MAC disease. Studies have demonstrated a correlation between silica exposure and mycobacterial infections,^5^^,^^6^ possibly because of impaired macrophage function^7^ and an increase in T helper cell 2 activity.^8^ This patient will be followed closely for signs of MAC disease.

The association between silicosis and engineered stone fabrication is concerning. A 2019 Center for Disease Control and Prevention report^9^ highlighted growing silicosis incidence in stone fabrication workers, noting that engineered materials often contain > 90% silica compared with < 45% in natural materials. An Australian study found silicosis in 12% of this workforce, suggesting possible underdiagnosis in the United States. Despite Occupational Safety and Health Administration regulations, implementation of protective measures has been inconsistent.^10^

In summary, this case underscores the critical importance of considering silicosis in patients with unexplained thoracic disease and stone fabrication history. The diagnosis in this case required a multidisciplinary approach. Even without evident pulmonary parenchymal disease, PET-avid lymphadenopathy and pleural abnormalities should raise concern for silicosis in patients with significant exposure. This case highlights occupational hazards in the stone fabrication industry and emphasizes the need for proper engineering controls and regular medical surveillance.

## Funding/Support

The authors have reported to *CHEST Pulmonary* that no funding was received for this study.

## Financial/Nonfinancial Disclosures

None declared.
